# Changing How Biologists View Flowers—Color as a Perception Not a Trait

**DOI:** 10.3389/fpls.2020.601700

**Published:** 2020-11-19

**Authors:** Jair E. Garcia, Ryan D. Phillips, Craig I. Peter, Adrian G. Dyer

**Affiliations:** ^1^Bio-Inspired Digital Sensing Laboratory (BIDS Lab), School of Media and Communication, RMIT University, Melbourne, VIC, Australia; ^2^Department of Ecology, Environment and Evolution, La Trobe University, Bundoora, VIC, Australia; ^3^Department of Biodiversity, Conservation, and Attractions, Kings Park Science, Perth, WA, Australia; ^4^Department of Ecology and Evolution, Research School of Biology, The Australian National University, Canberra, ACT, Australia; ^5^Department of Botany, Rhodes University, Grahamstown, South Africa

**Keywords:** mimcry, color modeling, orchid, signal detection, pollination, honeybee

## Abstract

Studying flower color evolution can be challenging as it may require several different areas of expertise, ranging from botany and ecology through to understanding color sensing of insects and thus how they perceive flower signals. Whilst studies often view plant-pollinator interactions from the plant's perspective, there is growing evidence from psychophysics studies that pollinators have their own complex decision making processes depending on their perception of color, viewing conditions and individual experience. Mimicry of rewarding flowers by orchids is a fascinating system for studying the pollinator decision making process, as rewarding model flowering plants and mimics can be clearly characterized. Here, we focus on a system where the rewardless orchid *Eulophia zeyheriana* mimics the floral color of *Wahlenbergia cuspidata* (Campanulaceae) to attract its pollinator species, a halictid bee. Using recently developed psychophysics principles, we explore whether the color perception of an insect observer encountering variable model and mimic flower color signals can help explain why species with non-rewarding flowers can exist in nature. Our approach involves the use of color discrimination functions rather than relying on discrimination thresholds, and the use of statistical distributions to model intraspecific color variations. Results show that whilst an experienced insect observer can frequently make accurate discriminations between mimic and rewarding flowers, intraspecific signal variability leads to overlap in the perceived color, which will frequently confuse an inexperienced pollinator. This new perspective provides an improved way to incorporate pollinator decision making into the complex field of plant-pollinator interactions.

## 1. Introduction

Those walking through a forest in early spring cannot help to notice the burst of biological activity evidenced by the wide array of different sounds, aromas and movement. However, due to the particularities and complex architecture of our own senses, the vast palette of color produced both by animals and plants will quickly catch our eyes and very likely drive our attention. Human fascination with colors presented by nature is old with formal writings on the topic dating back to Aristotle, one of the earliest naturalists, who pointed out that “*Whatever is visible is color and color is what lies upon what is in its own nature visible*” (Aristotle, 1970). Whilst modern color science separates the philosophical aspects of color theory from its physical nature, there still remains several ways to interpret color: either as a purely physical property of objects, as a subjective experience of the observer; or, by acknowledging that the physical aspects of color drive the subjective experience of the observer (Hatfield, 2003). Whilst the study of color in ecological and evolutionary contexts are currently mainly driven by purely physical aspects, i.e., treating color as a trait, it is potentially important to also consider the perceptual aspects of color vision to better understand how animals use this information to drive their behavior (Bruce et al., 2003).

The evolution of flower coloration has become a recurring research topic in biology, and color is often used as measurable trait for understanding different plant-pollinator interactions. We now recognize that many animals see the world differently to us, as suggested by data on the spectral sensitivity of their photoreceptors (Kemp et al., 2015). For example, many hymenopteran pollinators are characterized by possessing a trichromatic visual system perceiving spectral radiation between about 300 and 650 nm (Peitsch et al., 1992), allowing them to perceive UV radiation invisible to us, but limiting their ability to discriminate long wavelengths, which we and at least some other primates can easily recognize as being “red.” On the other hand, most birds have a tetrachromatic visual system that is often sensitive to ultraviolet and long wavelength radiation from about 350 to 700 nm (Hart and Hunt, 2007), allowing them to perceive spectral and non-spectral color stimuli (Stoddard et al., 2020). These differences suggest that different animals, including humans, very likely perceive the same object differently and as such all interpretations of a color signals should be made considering the specific characteristics of the visual system of the receiving animal (Cuthill et al., 2017).

In its most basic definition, the term trait is used to describe a measurable feature at the individual level, and as floral color can have a large impact on the fitness of a plant, it is considered a key functional trait (Violle et al., 2007; Phillips et al., 2020). When applied to plants, such as comparative studies testing for an association between colors and pollinator groups, some authors refer to color through visual attributes defined by human perception, such as brightness, saturation, and hue (Smith, 2014; Reverté et al., 2016). Whilst the use of these attributes has provided interestingly insights into the distribution of plant colors along spatial gradients (Gray et al., 2018), and its association with biotic and abiotic factors (Dalrymple et al., 2015, 2020; Reverté et al., 2016), it is still unclear if color attributes applicable to human vision are universal among animals and relevant for all species. Brightness, for example, is a confound to color perception (Kelber et al., 2003) and in primates during the early stages of visual processing the chromatic and achromatic information are separated (Livingstone and Hubel, 1988; Nassi and Callaway, 2009) into the magnocellular and pavocellular pathways. It is only latter that these pathways are integrated using multiple stages in the primate brain to enable the dynamic color perception including brightness that humans have (Nassi and Callaway, 2009). Currently there is no definitive proof that any non-primate animal processes brightness as a dimension of color vision, and thus using human perception to define traits of flowers that are not pollinated by primates is highly questionable. Therefore, there is a need to understand how pollinators perceive color signals.

With the exception of a few recent studies in plant-insect interactions (Shrestha et al., 2019), the perceptual aspect of vision, the brain's interpretation of a physical color signal (Cornsweet, 1971), is rarely considered in animal color studies (Endler and Mappes, 2017). Reasons for this are the scarcity of data on the complex relationship between the neurophysiological processing of color signals and behavioral responses triggered by these. With the exception of humans, the European honeybee (*Apis mellifera*) is the only animal model for which there is currently sufficient data allowing us to model the complex and dynamic perceptual aspect of color vision in real-world scenarios (Dyer, 2012).

Bees and other pollinating insects live and navigate in complex and constantly changing environments, where they have to continuously process visual information from target and distractors to make decisions, often several times per second (Spaethe et al., 2001). To better understand how a bee may perceive color information in different ways depending upon the context in which colors are encountered, it is important to (i) understand how color stimuli are sensed and stored in memory by a visual system, and (ii) that evidence shows that the reliability with which color information can be recalled from memory is dependent upon individual experience (Dyer, 2012). Thus, a bee cannot be regarded as an ideal observer with perfect acuity, memory and color discrimination capabilities. Instead, under a Darwinian framework, bees should be regarded as an animal acting for its own survival based on the sensory processing capabilities it has evolved.

Considering the effect of memory on color perception, signals from a stimulus are initially processed at a photoreceptor level by integrating spectral reflectance, illumination and relative photoreceptor sensitivities (Chittka, 1992; Vorobyev and Osorio, 1998; Spaethe et al., 2001; Kemp et al., 2015). When two differently colored stimuli are viewed side by side at exactly the same time a very precise color judgement can be made. This is termed simultaneous color discrimination and is analogous to when we want an exact paint or fabric match to a known model color, so we take a sample to view side by side with any potential candidate color. However, if we are required to make an evaluation of a model color to a sample that is spatially separated then the information captured by photoreceptors must be coded to memory and then when a subsequent comparison is made the color must be retrieved from memory to enable a judgement of whether the colors are indeed the same. This is termed successive color discrimination, and in both humans (Newhall et al., 1957) and honeybees (Dyer and Neumeyer, 2005) color judgements with successive viewing conditions are significantly poorer than when made simultaneously.

Regarding the effect of individual experience on color perception, psychophysics experiments on honeybees (Giurfa, 2004; Reser et al., 2012), bumblebees (Dyer and Chittka, 2004), and hawkmoths (Kelber, 2010) shows that the accuracy with which an individual insect can make such color judgements is dependent upon the level of experience with respective stimuli. Specifically, if an insect has only experienced one type of rewarding model color, which is termed *absolute conditioning*, color discrimination is subsequently relatively coarse when presented with similar alternative colors in a test. However, if an individual insect has the opportunity to learn a rewarding model color relative to a similar distractor, then learning occurs which results in changes in the brain and the enablement of long term memory (Dyer and Garcia, 2014; Sommerlandt et al., 2016).

Effects of memory and experience on color perception can be quantified by means of a function predicting the probability of accurate discrimination based on color similarity between two stimuli (von Helversen, 1972). Such a function has been formally derived from behavioral data (Dyer and Neumeyer, 2005) for honeybees and bumblebees considering simultaneous viewing condition (Garcia et al., 2017) and absolute conditioning (Garcia et al., 2018), but can also be formulated for successive color discrimination from behavioral data (Dyer and Neumeyer, 2005).

**Figure 1:**
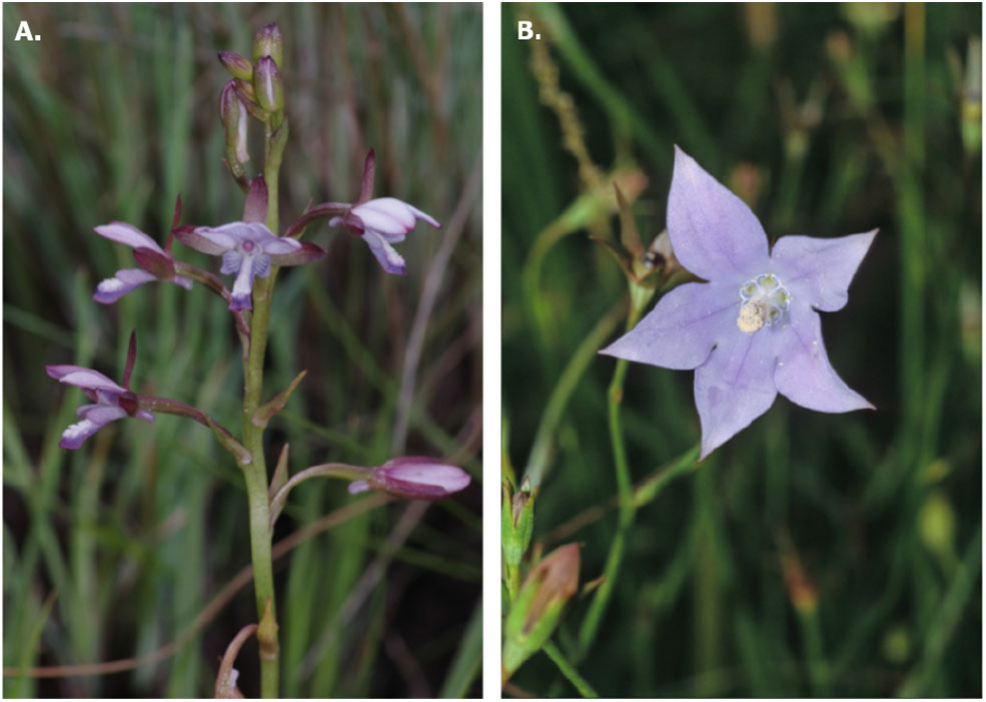
Flowers used in our study to understand pollinator decision making depending upon experience and viewing conditions. **(A)** Rewarding *Wahlenbergia cuspidata* (Campanulaceae) with mean flower size of 13.5 mm (Brehmer, 1915), and **(B)** rewardless *Eulophia zeyheriana* (Orchidaceae) with mean flower size of 8.4 mm (Rolfe, 1913). Photos by C. Peter.

If color rather than spectral reflectance is a functional trait, the predicted accuracy of discrimination between two stimuli should be unaffected by context and/or memory as the observer judgement of the difference should be based solely on the stimulus' physical properties. Rejection of this null hypothesis would suggest that perception prevents the generalization of conclusions based on purely physical aspects of color signals. A biological system for testing this hypothesis is that of deceptive orchids where a non-rewarding species closely resemble a rewarding flower (Peter and Johnson, 2008; Jersáková et al., 2016). In this scenario, resemblance between mimic and model should be close enough that pollinators are sometimes unable to reliably discriminate between them (Jersáková et al., 2016). Specifically, here we use published data from Peter and Johnson (2008) on petal color from the mimic *Eulophia zeyheriana* (Orchidaceae) and the rewarding flower *Wahlenbergia cuspidata* (Campanulaceae) (Figure 1) to test this hypothesis. In the absence of color discrimination data for the *Lipotriches* bee pollinating these species, we used color discrimination data from *Apis mellifera*, a model hymenopteran pollinator, as it is known that trichromatic color vision is phylogenetically conserved in bees (Briscoe and Chittka, 2001). Whilst the precise effect of color similarity on discrimination accuracy may differ between species, data from Australian and Neotropical singless bees (Garcia et al., 2017), and more recently pollinator flies (Hannah et al., 2019), suggest that color discrimination by insects can be accurately described by continuous functions of different shape. So whilst the precise color discrimination capabilities of *Lipotriches* may differ from those observed in *Apis*, data from the latter species serves as a valid example of the general model describing the effects of cognition and viewing condition on color discrimination as theoretically predicted by von Helversen (1972).

Accuracy to discriminate between two stimuli based on their color similarity can be used as an indirect measurement of task's difficulty. For example, an accuracy of 0.75 means that there is a chance of 0.25 for a bee to make an error. In other words, a bee will fail to discriminate a stimulus from a distractor about once in every four choices. Under our null hypothesis, the probability of discrimination between the mimic and model flower colors should remain at the same level independent of experience and viewing conditions: i.e., the probability of discriminating two stimuli based on their color difference should be independent of conditioning and viewing conditions. As an alternative hypothesis we propose that an acquired tolerance to “perceptual noise” arising from color variability on petals of the rewarding species (Garcia et al., 2018) affects the probability of a pollinator accurately discriminating between rewarding and mimic flowers. When a bee searches for a target it should be able to detect and discriminate it among a set of options, potentially including non-rewarding distractors. However, the color signal produced by rewarding “target” flowers is also variable and likely discriminable by bees (Paine et al., 2019). Therefore, a pollinator should balance the probability of rejecting a correct flower as a result of only accepting a narrow range of color variants of their target, i.e., increase their possibility of a Type I error. To decrease the chances of committing Type I errors, a bee could increase its tolerance to accept a wider range of the target's color variants. This solution, however, would then increase its chances of accepting non-rewarding mimics resulting in an increase of its Type II error (Endler and Mappes, 2017).

Lichtenberg et al. (2020) presented a theoretical model explaining the complexity of this scenario using univariate probability density functions (PDFs) to describe the effects of signal variability in both mimic and rewarding species. This is an interesting approach as PDFs better describe the distribution of flower colors in the wild in similar way to that encountered by a foraging bee. The area where two PDFs overlap creates a “confusion” region: signals falling within this area will be ambiguous and potentially difficult to discriminate by a pollinator as it could correspond to either the mimic or an infrequent color signal variant of the rewarding species (Lichtenberg et al., 2020). Here we apply and extend this theoretical model to color signals of wild plants to test if tolerance to perceptual noise, resulting from an increase in discrimination ability through experience and bee pollinator viewing conditions, is a likely explanation to the success of mimic orchid species.

## 2. Materials and Methods

### 2.1. Case Model: Flower Mimicry in Floral Deception

To test our null hypotheses of whether color signals are a constant trait rather than variable perception if values change depending upon bee viewing conditions, we used reflectance data from *Wahlenbergia cuspidata* and *Eulophia zeyheriana* published by Peter and Johnson (2008) (Figure 1). *E. zeyheriana* is a terrestrial orchid that is restricted to grasslands of the Drakensberg Mountains of the summer rainfall region of South Africa (Johnson and Bytebier, 2015). While the flowers are self-compatible, a pollen vector is required for effective pollination to occur. *E. zeyheriana* (Orchidaceae) is pollinated by males of a single undescribed species of *Lipotriches* bee (Halictidae). For a human observer, the rewardless flowers closely resemble those of the co-flowering nectar producing species *W. cuspidata* (Campanulaceae) (Figure 1). At these sites *W. cuspidata* is a major food source of the *Lipotriches* bees. The bees are thought to be attracted to *E. zeyheriana* through their similarity in color and overall floral shape to *W. cuspidata*. Both species have prominent petals that appear blue-violet to the human eye. The pollen presenter in *W. cuspidata* is white to the human eye and is similar in color to the white papilose area of the labellum of *E. zeyheriana*. While male bees do not collect pollen, the white area of the labellum of *E. zeyheriana* may be important for mimicking the overall floral pattern of *W. cuspidata*. Measurements of spectral reflectance revealed that the petals of both species are similar and located in the blue-UV segment of the hexagon model of bee vision (Peter and Johnson, 2008). An experiment where flowers were painted with a UV absorbing mixture revealed that flowers became less attractive to pollinators when they did not reflect UV, suggesting that floral color plays an important role in pollinator attraction. Scent is unlikely to be used as a luring cue as bees show no response to scent extracts of *Wahlenburgia* flowers (Welsford and Johnson, 2012). Further, reproductive success of the orchid was greater in close proximity to the model species (Peter and Johnson, 2008).

### 2.2. Absolute and Successive Viewing Color Discrimination Functions

We modeled color discrimination functions for *Apis mellifera* from isoluminant “blue” and “yellow” stimuli considering successive viewing and absolute conditioning using data from behavioral experiments (Dyer and Neumeyer, 2005; Garcia et al., 2018). Both functions describe the probability of accurate discrimination for increasing color differences, here expressed as distance in the hexagon space (Chittka, 1992), by means of a non-linear expression. Functions were fitted using a least-squares regression using the methods by Garcia et al. (2017) to model the color discrimination function for this species when stimuli were observed simultaneously. A separate function was modeled when color discrimination occurs under absolute conditioning; i.e., when bees learn the target stimulus in the absence of a distractor.

We fitted a non-linear mixed effect model using the package nlme for the R environment for statistical computing to produce the successive discrimination functions for the “blue” and “yellow” color stimuli. As the response variable we used the proportion of correct choices made by *n* = 5 and *n* = 4 bees when discriminating a reference stimulus from a set of nine different blue and yellow distractors, respectively varying in color similarity to the reference. See Dyer and Neumeyer (2005) for a complete description of the behavioral experiment and stimuli. Color dissimilarity between each reference/distractor pair, expressed as Euclidean distance in the hexagon color space (Chittka, 1992), was used as an independent variable in the model. Bee ID number was included as a random term in each model to account for the multiple measurements collected from each individual bee.

For the absolute discrimination behavioral experiment, a total of six different color stimuli were tested, comprised of three samples from the “yellow” and “blue” stimulus sets. Experimental data showed the same behavioral response from bees to larger color differences, so only three stimuli pairs were tested for each color. To ensure a robust fit, responses from the *n* = 9 tested bees to the six stimuli were pooled and used as response variable, so no random term was included in this model.

If color distance represents a measurable trait, the same mathematical function can be used to describe the relationship between color difference (Δ*C*) and probability of accurate discrimination (π) under absolute conditioning and successive viewing conditions. We formally tested this hypothesis by initially fitting a three (Equation 1) and four (Equation 2) parameter logistic functions to each data set, and subsequently used a likelihood ratio test (LRT) to compare between the two models. If the LRT test was not significant for an α = 0.05, we selected the simpler function following standard model selection procedures (Faraway, 2006). Under the null hypothesis, we expected that the two datasets can be modeled by the same type of function.

(1) $$\begin{array}{l} \pi =\frac{M_{o}K}{Mo+\left(K-Mo\right)\exp\left(-r\cdot \Delta C\right)} \end{array}$$

(2) $$\begin{array}{l} \pi =\frac{Mo+\left(K-Mo\right)}{1+\exp\left(\frac{xmid-\Delta C}{r}\right)} \end{array}$$

In the three parameter function (Equation 1), *K* defines the upper limit of the function, *M*_*o*_ represents the Δ*C* value at which the function begins to increase rapidly, and *r* gives the increment rate. In the four parameter model, *K* and *M*_*o*_ indicate values of the upper and lower asymptotes of the function, respectively; *r* describes magnitude of the increment rate, and *xmid* determines the value of Δ*C* corresponding to the first inflection point of the curve (Garcia et al., 2017).

Even if the functions modeling color discrimination under absolute conditioning and successive viewing have the same number of parameters, it is possible that the coefficients shaping each function differ thus suggesting a different relationship in each case. Mechanistically, such changes would reflect how the brain of honeybees changes and develops long term memory depending upon conditioning (Sommerlandt et al., 2016). Under the null hypothesis, probability of accurate discrimination predicted by both functions should not be significantly different in spite of shape differences; therefore, we also tested for equality between the mean probability of accurate discrimination predicted by the absolute and successive functions using a bootstrap test for equality of means (Hall and Hart, 1990; Efron and Tibshirani, 1993). For completeness, we also compared the shape of absolute conditioning and successive viewing conditions against the function for simultaneous discrimination published by Garcia et al. (2017). All bootstrap tests were performed with 100,000 samplings with replacement.

### 2.3. Spectral Measurements

For full methodology see Peter and Johnson (2008). However, briefly, the reflectance spectra of floral parts of the two species were measured using an Ocean Optics S2000 spectrophotometer (Ocean Optics, Dunedin, FL, USA), coupled to an Ocean Optics Mini-D2T light source. We measured spectra of *n* = 25 adaxial petal surfaces of separate flowers for both *E. zeyheriana* and *n* = 26 *W. cuspidata* flowers, as well as *n* = 22 point samples of the prominent white papillose area of the labellum of *E. zeyheriana* and *n* = 22 samples of the pollen-covered pollen presenter of male-phase *W. cuspidata* flowers. Raw spectral data were binned into 10 nm intervals between 300 and 650 nm using the piece wise cubic Hermite interpolating Polynomial routine for Python 3.7. Spectra was subsequently modeled in the hexagon color space by Chittka (1992) assuming an average green leaf as adaptation background (Bukovac et al., 2017) and an illumination typical of a clear midday open sky in the Northern hemisphere (Judd et al., 1964) expressed as photon flux using custom code written for Matlab release 2017 (The Mathworks, USA).

### 2.4. Color and Statistical Modeling

The particular conditioning of a pollinating bee and target viewing conditions, either simultaneous or successively, determines the minimum color difference it requires to accurately discriminate between two samples (Dyer and Chittka, 2004; Giurfa, 2004). However, color variability in the observed flowers determines the frequency by which the pollinator will encounter a flower pair whose color difference is low enough that they cannot be reliably discriminated between.

**Figure 2:**
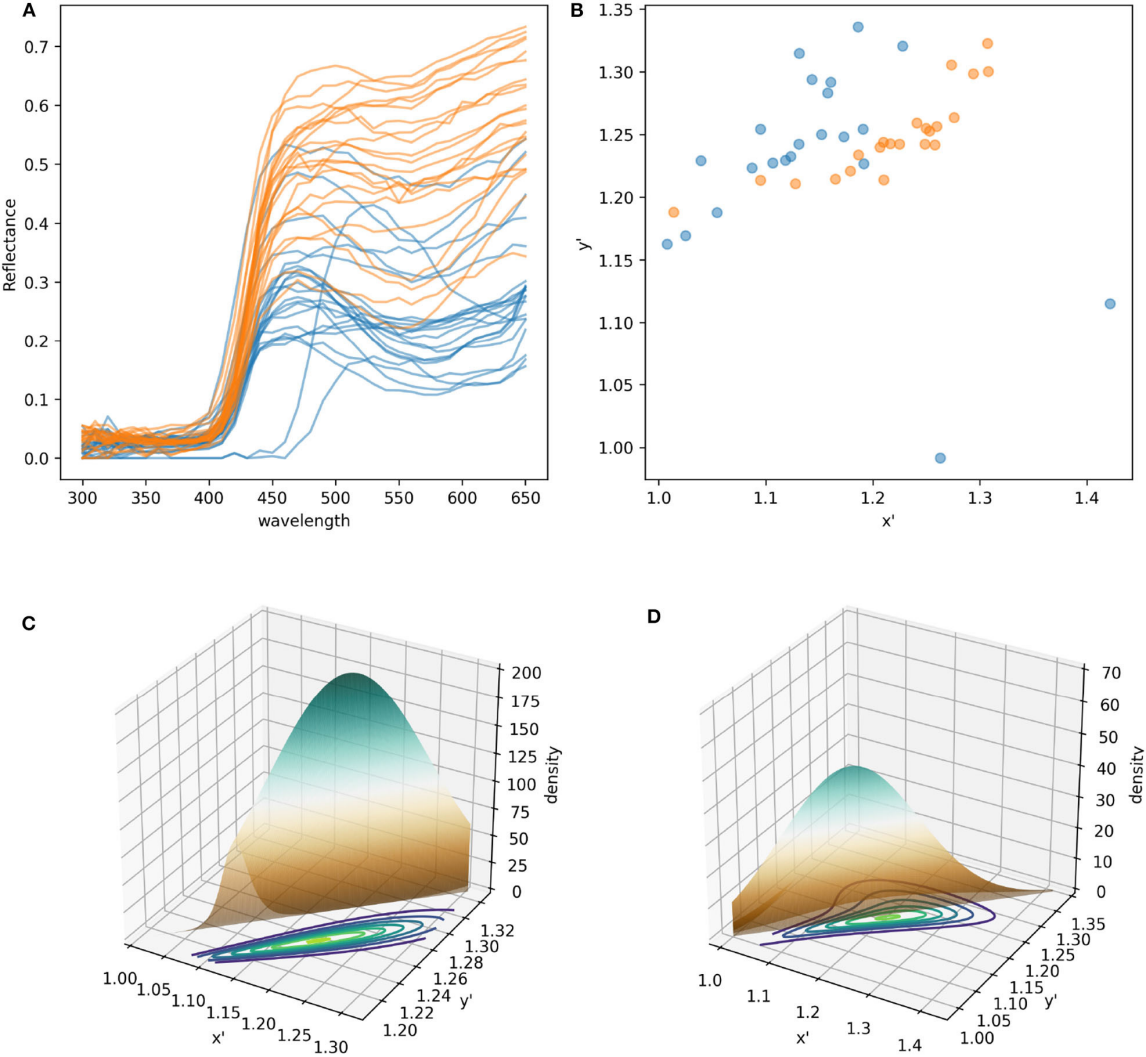
Modeling and statistical analysis of color variability in flower samples. **(A)** Reflectance profiles of pollen grains from *W. cuspidata* (blue) and labella region of *E. zeyheriana* (orange). **(B)** The same samples following modeling in the hexagon color space and translating the original x-y coordinates to an alternative x′ y′ coordinate system to ensure that all values are positive. Surfaces describing the probability density functions corresponding to the orange **(C)** and blue **(D)** cloud of points. Note how differences in variability are reflected in different maximum density values in the two PDFs as indicated by the surface's maximum height. Contour maps of **(C,D)** are two-dimensional representations of their respective surfaces. On these maps each contour represents different density values in analogous way to physical maps that use contours to represent altitude variations in the landscape.

Color signals produced by flowers and perceived by trichromatic pollinators, such as most bees (Briscoe and Chittka, 2001), are modeled as bivariate variables. More specifically, the spectral profile making up the color signal can be modeled in a two dimensional space (Shrödinger, 1970), such as the Maxwell triangle (Neumeyer, 1980), hexagon color space (Chittka, 1992), or other alternative models each with their own set of assumptions (see Renoult et al., 2017 for a review). For example, modeling the different spectral measurements collected from pollen of *W. cuspidata* and labella of *E. zeyheriana* produces two clouds of points whose shape, distribution and sparseness correspond to differences in their spectral profiles (Figures 2A,B).

The colors more frequently observed on each species will be clustered together in the same region of color space. If variability is low, most samples will be located in a small area of color space resulting in PDF high density (*d*) values. On the contrary, if variability is high, colors will be distributed in a wider area of color space resulting in a lower density. In a two dimensional space PDFs are not represented as curves, but as mound-shaped surfaces where the breadth and height of the peak is given by density of the most frequently observed loci (Figures 2C,D).

The most commonly used multivariate distribution to model two dimensional data is the (bivariate) joint normal distribution where the two variables are described by the same univariate distribution. This condition, however, is rarely observed in natural samples as flower colors in plant populations tend to be clustered in particular areas of color space (Chittka and Menzel, 1992; Dyer et al., 2012; Shrestha et al., 2014). A solution to model complex PDFs is modeling the joint cumulative distribution function for two continuous variables (marginals), each described by a different distribution, and their dependence structure independently through a copula (Genest and Favre, 2007).

Marginals and copulas describing the PDFs for petals, pollen presenter and labella of *W. cuspidata* and *E. zeyheriana* were fitted by maximum likelihood employing the package Vinecopula (Nagler et al., 2019) for the R language and environment for Statistical Computing (R Core Team, 2020). Marginals were fitted by maximum likelihood using the package fitdistrplus (Delignette-Muller and Dutang, 2015) for R, and tested for goodness of fit using the Anderson-Darling test available in the ADGofTest package (Bellosta, 2011) for R.

### 2.5. Likelihood of Discrimination in the Presence of Color Noise

In a symmetrical PDF, such as a bivariate normal distribution, frequency of observed loci could be predicted from the analytical expression describing this distribution as done in most parametric multivariate analysis techniques (Johnson and Wichern, 2007). However, such an approach cannot always be implemented when PDFs are modeled by copulas. To identify typical, less frequent and rare colors from the different plant species, we generated 100,000 random samples from their corresponding PDFs and calculated their density using functions available in the package copula (Kojadinovic and Yan, 2010) version 1.0-0 for R. For each species we subsequently obtained density values corresponding to the 0.9, 0.55, 0.45, 0.25, and 0.15 probability quantiles. As larger density values correspond to a higher probability, we assigned as typical colors loci whose density values were higher than the 0.9 quantile; less frequent colors as those whose probability of occurrence is between 0.45 and 0.55; and as rare, colors with density values corresponding to a probability between 0.15 and 0.25.

If probability density functions can tell us the likelihood of observing a flower of any given color for a species, discrimination functions will predict the probability with which given flower colors can be discriminated given a bee's experience and viewing conditions. We can use both of these functions to predict if pollinating bees can perceive as being different typical, less frequent and rare flower colors. To answer this question, we applied the same sampling method for each species PDF and classified them as typical, less frequent or rare based on their density values. Then, we calculated the Euclidean distance between loci pairs belonging to each group and obtained their mean distance, and used functions to obtain their predicted probability of discrimination.

We used a similar approach to predict the probability with which a bee can discriminate between likely colors of two different species. We generated random loci from the respective PDFs, and calculated the Euclidean distance between each loci pair. We then used the color discrimination functions to obtain the probability of accurately discerning between each stimulus pair considering absolute conditioning, successive and simultaneous viewing conditions.

## 3. Results

### 3.1. Color Discrimination Function Under Successive Viewing Conditions

We initially tested if a function with four parameters was necessary to obtain a better fit of data describing discrimination under successive viewing conditions, or if a simpler alternative with one less coefficient could provide the same fit using a likelihood ratio (LRT) test. We found no significant difference in the goodness of fit provided by a three or four parameter logistic function for either the blue or yellow stimuli ($\chi_{blue}^{2}=0.161,P=0.688;\chi_{yellow}^{2}=1.73,P=0.188$), so the former function was used to model the two data sets as it is mathematically simpler.

For the “blue” and “yellow” color stimuli we initially fitted a non-linear model including a random term for each one of the three parameters and alternative reduced models including only fixed terms for each term, followed by LRT comparisons. This method allowed us to identify which of the three parameters was significantly varying across individuals so that it should be included as a random term in the final model (Pinheiro and Bates, 2000).

**Figure 3:**
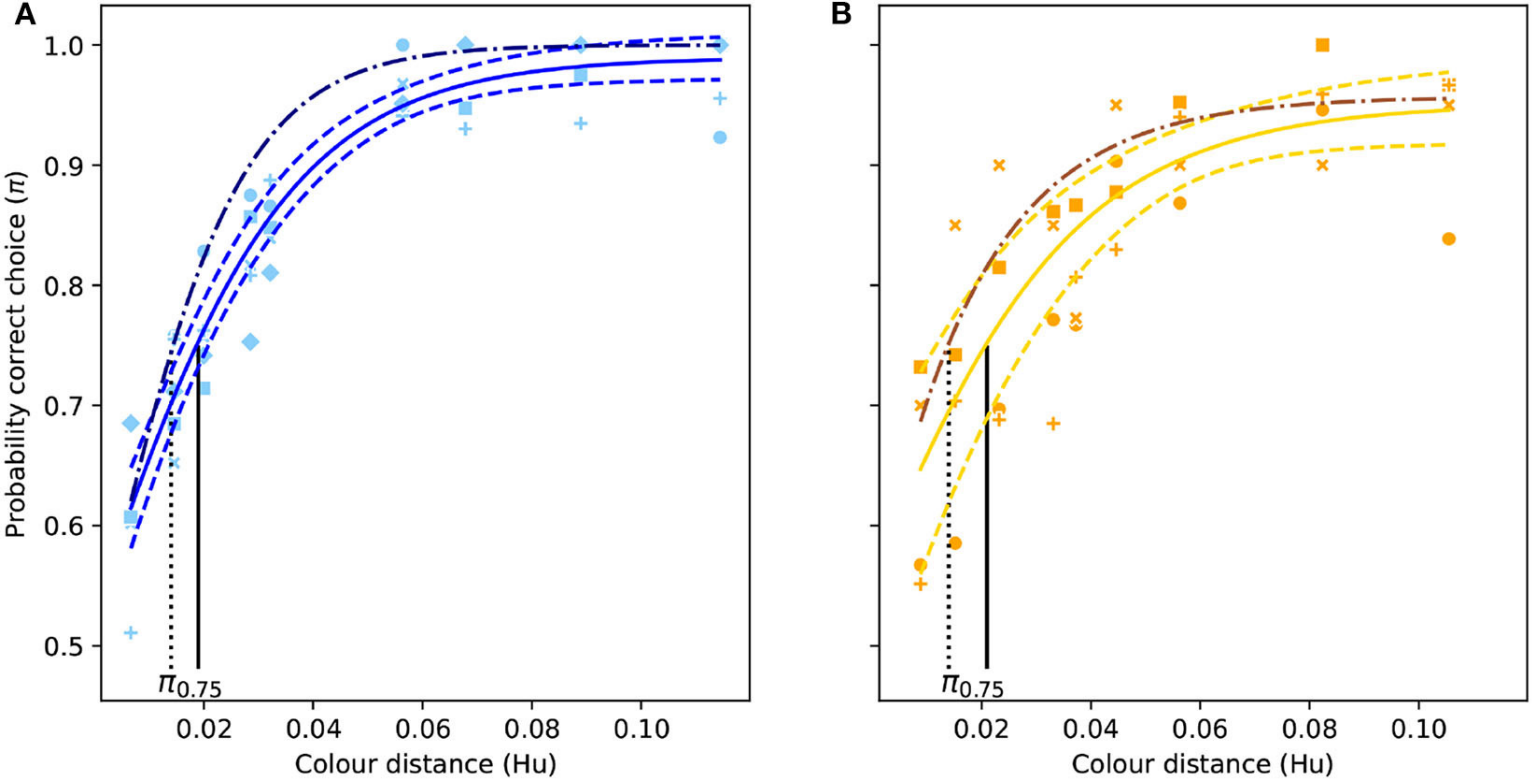
Color discrimination functions (solid colored lines) for deferentially-conditioned *Apis mellifera* when successively viewing different isoluminant “blue” **(A)** and “yellow” stimuli **(B)** varying in color similarity here expressed as Euclidean distance in the hexagon model (Hu) for hymenopteran vision (Chittka, 1992). Vertical black line indicates the color distance at which a bee observer will discriminate between a pair of color stimuli with a probability of 0.75 (π_75_). Dashed lines represent the 95% confidence intervals for their respective function, and the dash dotted line represent the color discrimination function for simultaneous viewing for the same stimuli (Garcia et al., 2017). Markers represent the proportion of correct choices made by individual bees.

For the “blue” and “yellow” stimuli functions the second term showed the highest variability across individuals so it was included as a random term in both models. Values for coefficients (*K, r, Mo*), defining the shape of each discrimination function are provided in Table 1, and a graphical representation of the two functions is given in Figure 3.

**Table 1:** Coefficients (*K, r, Mo*) defining the shape of a three parameter logistic curve (Equation 2) describing the probability of accurate color discrimination by *Apis mellifera* when successively viewing “blue” and “yellow” stimuli increasing in color dissimilarity.

	**K**	**r**	**Mo**
Blue	0.990	53.8	0.528
	(0.969, 1.01)	(45.3, 66.3)	(0.474, 0.571)
Yellow	0.951	47.2	0.555
	(0.913, 0.989)	(35.7, 69.5)	(0.420, 0.651)

*Values in parentheses are the 95% confidence intervals for each coefficient*.

**Figure 4:**
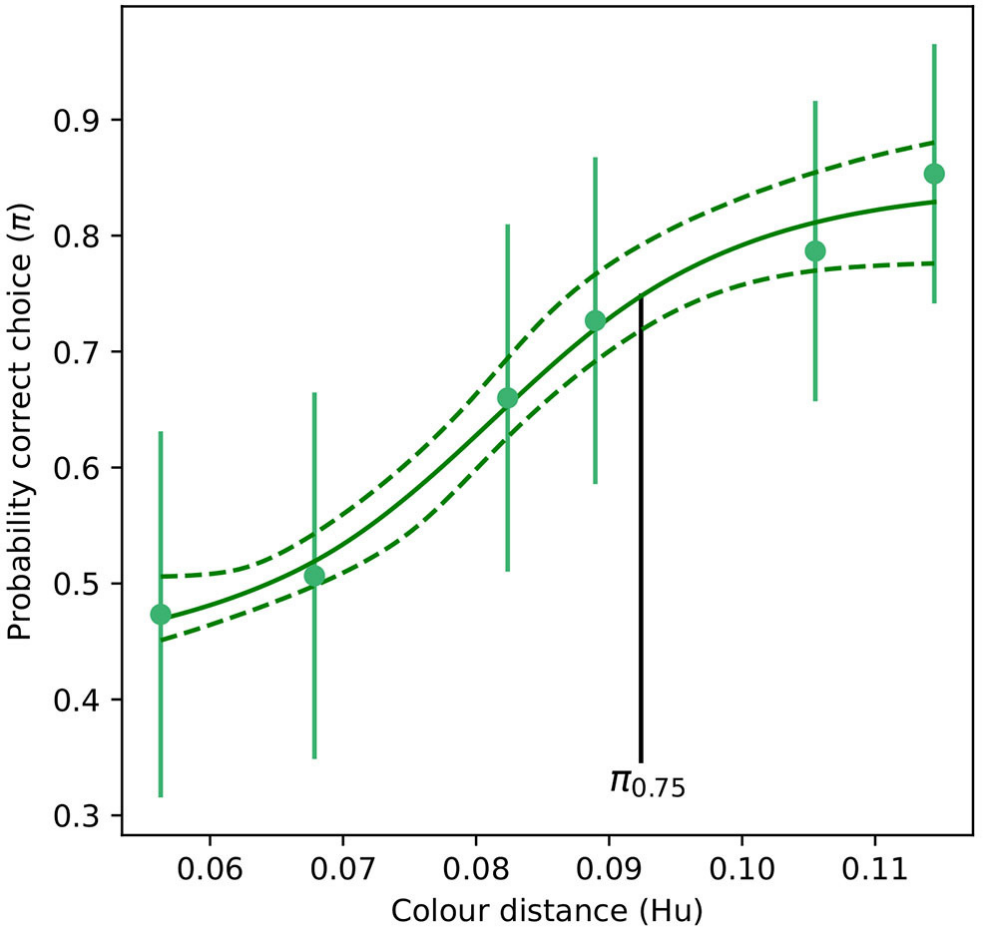
Color discrimination function for inexperienced *A. mellifera* under absolute conditioning. The vertical black line indicates the color distance at which a bee observer will discriminate between a pair of color stimuli with a probability of 0.75 (π_75_). Note that for an inexperienced bee, larger color distances are required to achieve the same probability of accurate discrimination than for the experienced forager modeled in Figure 3. Dashed lines indicate the 95% confidence intervals for their respective function. Markers represent mean responses from *n* = 9 bees. Whiskers indicate the standard error of the mean proportion of correct choices.

For the absolute color discrimination function, a four parameter logistic model provided a significantly better fit than the simpler, three parameter alternative (χ^2^ = 4.69, *P* = 0.030) so the former was used to fit the data. Coefficients describing the absolute color discrimination function for *A. mellifera* are provided in Table 2, and a graphical representation of the function is given in Figure 4.

**Table 2:** Coefficients (*Mo, K, xmid*, and *scal*) defining the shape of a four paramter logistic curve (Equation 1) describing the probability of accurate color discrimination by *Apis mellifera* when discriminating “yellow” and “blue” stimuli following absolute conditioning.

	**K**	**Mo**	**xmid**	**scal**
Absolute	0.842	0.441	0.081	0.009
	(0.766, 0.919)	(0.377, 0.505)	(0.076 0.086)	(0.004, 0.015)

*Values in parentheses are the 95% confidence intervals for each coefficient*.

Mean probability of accurate discrimination predicted by the absolute conditioning and successive viewing conditions were significantly different from each other (*t*_*Ho*_ = −22.0, *P* < 0.001). Likewise, significant differences in predicted accuracy were observed between the absolute and successive functions (*t*_*Ho*_ = −23.5, *P* < 0.001), and between successive and simultaneous conditions (*t*_*Ho*_ = −16.5, *P* < 0.001). This result evidences a significant effect of experience and memory on the perception of color differences by *A. mellifera*.

### 3.2. Effect of Color Difference and Variability

**Figure 5:**
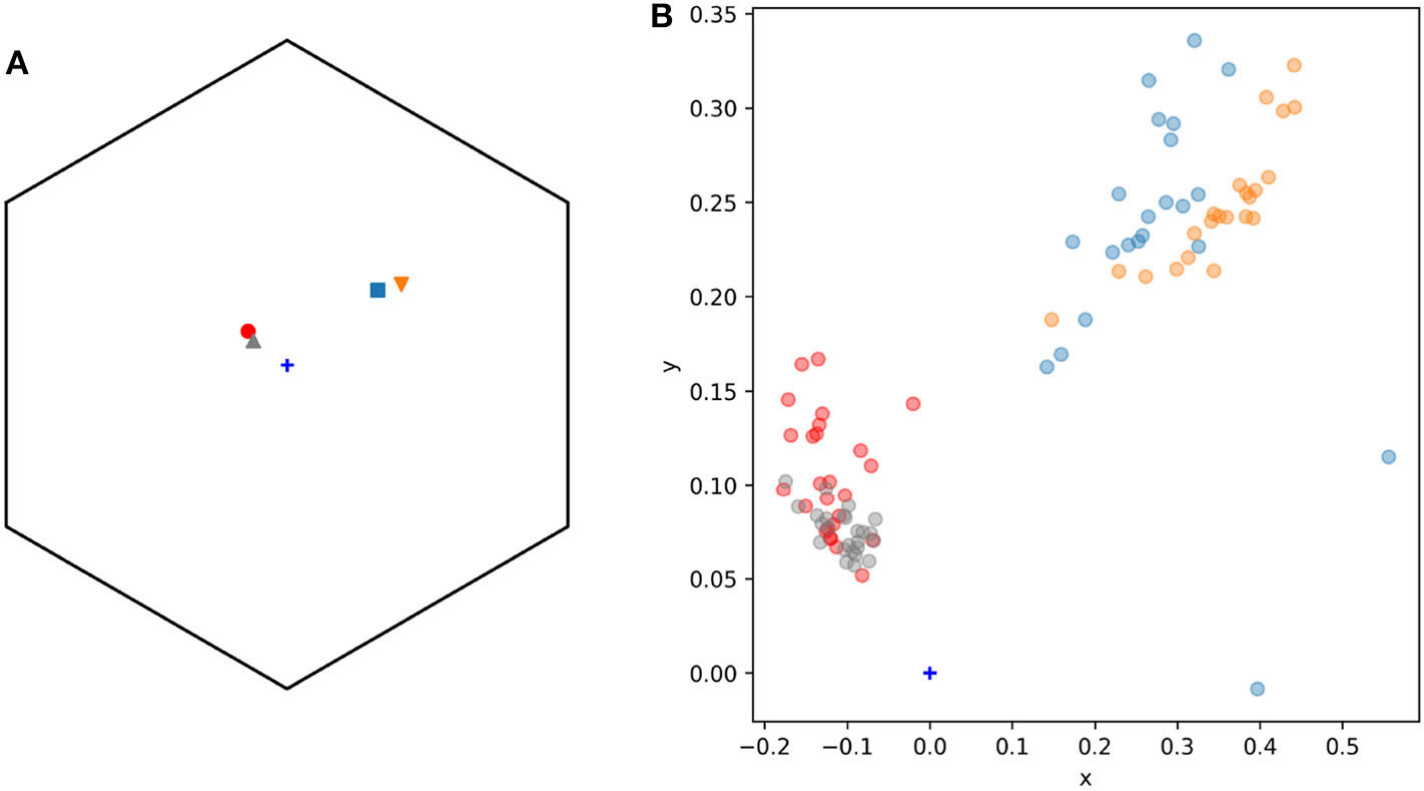
General view **(A)** and detail **(B)** of the *n* = 95 spectral samples used to model adaxial lateral petals (*n* = 25, triangular markers) and labella (*n* = 22, inverted triangles) of the mimic orchid *Eulophia zeyheriana*, and adaxial petals (*n* = 26, circle markers) and pollen (*n* = 22, square markers) of the rewarding species *Wahlenbergia cuspidata*. **(B)** Shows a detail of **(A)** highlighting color variability in sampled regions. Marker colors were selected to ease visualization: gray (lateral petals *E. zeyheriana*), red (petals *W. cuspidata*), orange (labella *E. zeyheriana*), and blue (pollen *W. cuspidata*). Color space's center is indicated by a blue cross marker.

The color of lateral petals of the orchid *Eulophia zeyheriana* and the sympatric rewarding species *Wahlenbergia cuspidata* appear blue to the human eye and fall between the blue and blue-UV sectors of the color hexagon (Figure 5). Consequently the “blue” discrimination function was used to model this system.

Mean color differences between all petals samples of *E. zeyheriana* and *W. cuspidata* was 0.056 (Hu) ±0.032 (standard deviation). Such a color difference cannot be discriminated by an inexperienced bee as the probability of accurately discriminating between such a stimuli is of 0.5. However, an experienced bee can discriminate between petals of the two species with an accuracy of 0.949 and 0.987 when comparing them in succession or simultaneously, respectively. Color differences between the labellum of *E. zeyheriana* and the pollen presenter of *W. cuspidata* are easier to differentiate by a bee. Mean color differences between these two stimuli is equal to 0.138 ± 0.074 (Hu), which can be discriminated by an inexperienced bee with an accuracy of 0.841. This value increases to 0.99 for an experienced bee comparing between these stimuli either successively or simultaneously. Altogether, the results indicate that the perception of a given color difference changes with experience of the pollinator and is also context dependent, hence perception prevents interpreting the color signal as a trait. Our results suggest that spectral reflectance data should always be interpreted in a specific context, thus rejecting the null hypothesis that color interpretation is independent from viewing conditions and experience; we thus proceed to test the color noise alternative.

**Figure 6:**
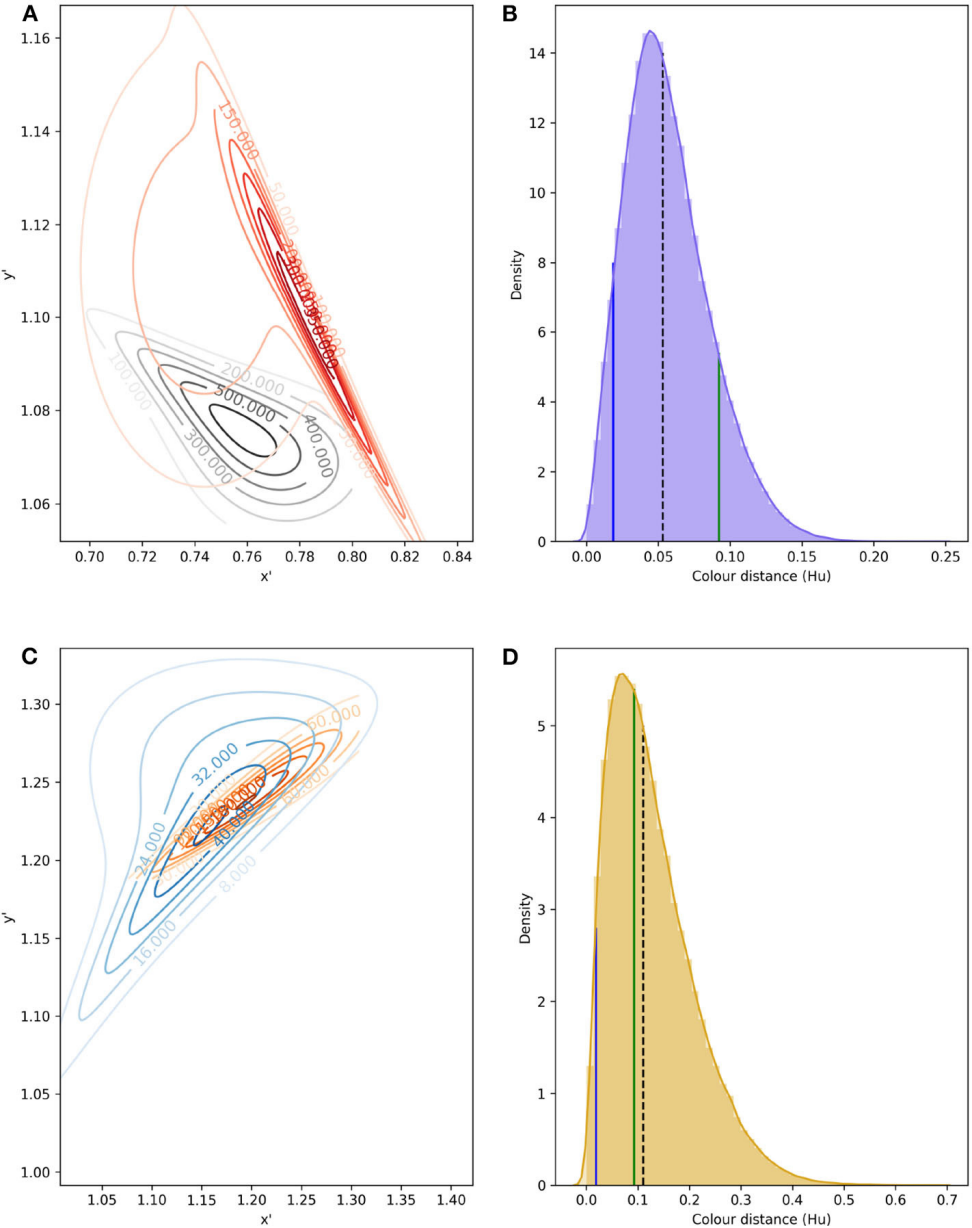
Confusion region in hexagon color space produced by the overlap of bivariate PDF describing variability in color signal produced by lateral petals of the orchid *E. zeyheriana* and petals of the rewarding flower *W. cuspidata* **(A)**; in addition to labella and pollen presenter from respective species **(C)**. Numbers on the contour lines indicate the various density values for each PDF, larger magnitudes represent areas of color space where most samples occur for each species. All loci were translated from their original hexagon x-y coordinates into a new set of coordinates x′ and y′ where all values are positive. Distribution of Euclidean distances obtained after drafting 100,000 random samples from petal **(B)** and labella/pollen distribution **(D)**. In **(B,D)**, black, dashed lines represent the median distance; solid, green lines indicate the color difference which can discriminated by an inexperienced bee with an accuracy of 75%; and blue lines indicate color distance for the required by an experienced forager to attain the same accuracy when observing both targets successively.

To model signal noise produced by color variability, bi-variate probability density functions (PDFs) were fitted to data from petals, pollen and labellum from rewarding and mimic, respectively, using copulas (Figure 6). Parameters defining each PDF are given in Table 3.

**Table 3:** Parameters for the different elements describing the probability density function (PDF) of color loci for the different flower parts considered.

**Stimulus**	**Component**	**Distribution**	**Parameter (SE)**
*W. cuspidata* petals	x′	Gamma	Shape = 491 (136)
			Rate = 659 (183)
	y′	Gamma	Shape = 1270 (353)
			Rate = 1150 (320)
	Copula	Tawn II 270°	Par 1=-9.13 (4.75)
			Par 2=0.244 (0.030)
*W. cuspidata* pollen presenter	x′	Gamma	Shape = 182 (54.9)
			Rate = 159 (48.0)
	y′	Weibull	Shape = 22.8 (3.86)
			Scale = 1.26 (0.012)
	Copula	Tawn II 180°	Par 1=3.61 (0.846)
			Par 2=0.490 (0.110)
*E. zeyheriana* lateral petal	x′	Normal	Mean = 0.761 (0.006)
			Std = 0.027 (0.004)
	y′	Normal	Mean = 1.08 (0.002)
			Std = 0.012 (0.002)
	Copula	Joe 90°	Par 1 = −2.15 (0.552)
*E. zeyheriana* labellum	x′	Normal	Mean = 1.22, (0.015)
			Std = 0.070 (0.011)
	y′	Gamma	Shape = 1370 (423)
			Rate = 1100 (339)
	Copula	Survival Gumbel	Par 1= 4.19 (0.764)

*Each PDF is defined by the univariate distribution of the values for x and y color coordinates in the hexagon space (marginals) and their join distribution modeled by a specific copula type. SE denote the standard error for each parameter value*.

Low color noise on petals of both species make typical, less frequent and rare colors more similar to each other and thus harder to discriminate by an insect pollinator. Indeed, mean color difference between typical and rare petal colors of *E. zeyheriana* and *W. cuspidata* is of about 0.025 and 0.051 Hu, respectively, which are unlikely to be discriminated by an inexperienced bee forager, here modeled using the discrimination function for absolute conditioning. However, an experienced pollinator would be able to discriminate between typical, less frequent and rare petal colors of *E. zeyheriana* with an accuracy between 80 and 90% when viewing them successively. This value increases to about 90% if colors are viewed simultaneously (Table 4). Larger color difference in petals of *W. cuspidata* makes discrimination between typical and less frequent colors more likely with a probability of an accurate discrimination higher than 0.9 for successive and simultaneous viewing conditions.

**Table 4:** Probability of a honeybee discriminating between pairs of typical (typ.), less frequent (lfq.) or rare (rar.) colors for various flower regions of *E. zeyheriana* and *W. cuspidata* under absolute conditioning, or when seeing targets successively or simultaneously.

**Species**	**Region**	**Pair**	**d**	**ΔC**	**Abs**	**Succ**.	**Simul**.
*E. zeyheriana*	Lateral petal	typ./lfq.	>590	0.025	0.500	0.806	0.873
		typ./rar.	300 ≤ d < 360	0.035	0.500	0.874	0.938
		lfq./rar.	100 ≤ d < 166	0.040	0.500	0.899	0.957
	Labellum	typ./lfq.	> 188	0.063	0.500	0.962	0.993
		typ./rar.	87 ≤ d < 109	0.094	0.765	0.985	0.999
		lfq./rar.	26 ≤ d < 45	0.100	0.759	0.986	1.00
*W. cuspidata*	Petal	typ./lfq.	> 250	0.051	0.500	0.937	0.982
		typ./rar.	90 ≤ d < 108	0.070	0.532	0.970	0.996
		lfq./rar.	32 ≤ d < 53	0.057	0.500	0.951	0.988
	Pollen	typ./lfq.	> 42	0.086	0.716	0.982	0.999
		typ./rar.	19 ≤ d > 23	0.135	0.841	0.989	1.00
		lfq./rar.	6 ≤ d < 10	0.146	0.842	0.990	1.00

*Color dissimilarity is expressed as mean distance in the hexagon space (*Δ*C) in all cases. Typical, less frequent and rare colors where determined from density (d) values of the PDF corresponding to each sample*.

Labellum and pollen color loci show higher variability than petals in both *E. zeyheriana* and *W. cuspidata*. Mean color difference between typical and rare colors of *E. zeyheriana* labella is ~0.06 Hu, whilst differences in pollen color of *W. cuspidata* is ~0.086 Hu. Whilst differences between labella cannot be discriminated with an accuracy higher than 50% by inexperienced foragers, our model predicts that the same observers would be able to discriminate between typical and less frequent pollen colors about 70% of the time. Experienced foragers are predicted to discriminate between typical and less frequent colors of labella and pollen with an accuracy higher than 90% when viewing these stimuli either successively or simultaneously (Table 4).

The confusion region produced by overlap of probability density functions (PDFs) from petals of *E. zeyheriana* and *W. cuspidata* suggests that the most frequent colors produced by each species roughly occupy the same area of color space (Figure 6). In a scenario where both mimic and rewarding species have a similar abundance, bees searching for the rewarding species will very likely find flowers of the mimic whose color is very similar to the most frequently displayed by rewarding flowers. Indeed, the median color distance observed between petals of the two species is of about 0.053 Hu, which cannot be discriminated by an inexperienced bee. However, both colors can be identified as being different with a probability of 0.94 by an experienced bee if both targets as seen successively, and with a probability of 0.98 if seen simultaneously. Furthermore, 87.6% of comparisons between the lateral petals of the orchid flowers and petals of the rewarding species are below the 75% discrimination threshold for an inexperienced bee, although this proportion falls to 7.2% when considering an experienced forager observing petals from the two species and to 4.3% when flowers are observed simultaneously. The range of color distances for petals of *E. zeyheriana* and *W. cuspidata*, along with their associated probability of accurate discrimination by either inexperienced or experienced forager bees, is presented in Table 5.

**Table 5:** Median and range of color distances (Δ*C*) observed after randomly drawing 100,000 samples from the PDF describing color variability of the petals and pollen of the rewarding plant *W. cuspidata*; and, lateral petals and labellum of its mimic orchid *E. zeyheriana*.

**Flower region pair**	**ΔC**	**π_*abs*_**	**π_*succ*_**	**π_*simul*_**
Lateral petal/petal	0.053	0.500	0.941	0.984
	(≈0, 0.244)	(0.500, 0.842)	(0.529, 0.989)	(0.500, 1.00)
Labellum/pollen	0.110	0.826	0.987	1.00
	(≈0, 0.679)	(0.500, 0.842)	(0.535, 0.989)	(0.502, 1.00)

*Third to fifth columns contain the predicted probability of discrimination for either absolute (π_abs_) conditioning, successive (π_succ_), or simultaneous (π_simul_) conditions corresponding to the reported color distances based on the discrimination functions in Figure 3*.

Labella and pollen presenter color in *E. zeyheriana* and *W. cuspidata*, respectively, show a higher variability than petal color as indicted by the maximum density values of their PDF (Figure 6). Such a variability results in larger median color differences between these targets (0.110 Hu) than those observed for petals, in spite of presenting a larger confusion region in color space (Figure 6). This increase is the result of a higher chance of observing less frequent labella and pollen presenter colors, thereby facilitating discrimination for both inexperienced and experienced foragers (Table 5). Indeed, 41% of color comparisons between labellum and pollen colors will be below the 75% accuracy threshold for an inexperienced bee, whilst 3.1% of these comparisons will be below this level when considering an experienced bee observing both stimuli successively. The proportion of comparisons below the accuracy threshold diminishes to 1.8% when stimuli are observed simultaneously.

## 4. Discussion

When using color distance, researchers seek to infer from this metric if a particular flower color signal has an effect on plant fitness. Whilst this metric can potentially describe differences in the physical nature of a color signal between flowers, it cannot predict unambiguously if such a difference is perceivable by a pollinator.

When answering questions about the behavioral response of an animal to perceived color differences, discrimination functions provide a more realistic prediction of what an animal may perceive and its response. Compared to morphological traits, such as shape, length, and width of advertising floral parts measured with precise instruments (Violle et al., 2007), the interpretation of color difference by an animal brain is frequently context dependent and thus not a trait. In the current manuscript, we show that considering absolute conditioning, an inexperienced bee would be able to discriminate a color difference of about 0.09 Hu with an accuracy of 75%. However, after a bee has acquired more experience it will be able to discriminate the same color difference with an accuracy of almost 100% (Figures 3, 4). As such, we show that for a given color distance it is more appropriate to discuss the likelihood that an inexperieced or experienced pollinator is deceived by the mimic, rather than using a single color distance threshold.

Functions defined by Equations (1) and (2) and corresponding coefficients (Tables 1, 2), allow for the construction of modeling tools describing color discrimination by honeybees considering multiple viewing conditions including absolute conditioning (Figure 4), and simultaneous or successive color discrimination (Figure 3). Significant difference in the predicted probability of accurate discrimination by respective functions for the same color distance rejects the null hypothesis of equality and evidences an effect of both experience and conditioning on perception of floral color difference by a pollinating bee. This result thus indicates that though a flower's reflectance spectrum can be considered as a functional trait in some circumstances (Dalrymple et al., 2020), its interpretation by the brain of a pollinator cannot. Therefore, the *color sensation* experienced by a bee is frequently context dependant, and as such, cannot be quantified and compared as other purely physical traits. Interestingly, the use of functions like those presented in Figures 3, 4 produce data from physical traits that are compatible with a signal detection theory (Endler and Mappes, 2017; Lichtenberg et al., 2020), which better predict bee behavioral responses when foraging in the presence of rewarding targets and non-rewarding distractor flowers.

Studies of plant-pollinator interaction can benefit from considering insect perception of flower color, as this can provide better explanations of the relationship between the spectral color signal as measured by a spectrometer and animal behavior. For example, Peter and Johnson (2008) concluded that the orchid *E. zeyheriana* is a non-rewarding mimic of the rewarding flowers of *W. cuspidata*, based on several lines of evidence, including their color difference. When considering petal color variability from the two plant species we predict that about 25% of flower comparisons made by an inexperienced bees will be easy to discriminate (Figure 6B), whilst an experienced bee viewing the same colors successively would make perceptual errors <5% of the time (Figure 6B). Considering simultaneous viewing conditions, a bee would almost never make a perceptual error (Table 5), further reinforcing the contextual nature of color as a perceptual signal. Thus, the color modeling provides insights into how bee pollinators contribute to the pollination system in a dynamic way.

Classically, deceptive flowers are thought to rely on inexperienced insect visitors for pollination (Jersáková et al., 2006). Whilst our modeling confirms that inexperienced bees are often unable able to discriminate between mimic and model species (Figure 4), bees can readily distinguish these colors after acquiring some experience in spite of memory limitations (Figure 3). Honeybees under differential conditioning can learn to discriminate colors following 15 (Giurfa, 2004) to 50 choices for very similar stimuli (Reser et al., 2012), and these differences form memory lasting for at least 48 h days after initial testing (Dyer and Garcia, 2014). In the wild, however, such a learning does requiring visiting some mimics. Whilst the precise number of visits to either rewarding or mimic species by *Lipotriches* bees is unknown, it is very likely that hundreds of visits are done in the wild at least to rewarding flowers, suggesting that learning likely occurs in natural settings.

Considering that experienced bees are likely to discriminate between mimic and rewarding species (Table 5), an alternative explanation for the repeated visitation to flowers observed in the *E. zeyheriana-W.cuspidata* system (Peter and Johnson, 2008) is that bees develop a tolerance to color variability as a means to maintain flower constancy toward the rewarding species. Color variation in *W. cuspidata* is large enough that less frequent and rare colors are easy to discriminate from the more typical flowers (Table 4). Thus, bees visiting the rewarding species should have to develop a tolerance to “color noise” in order to identify the various colors displayed by flowers of this species (Figure 5). As color variability of the mimic is lower than the rewarding species, bees are likely to accept flowers of the mimic as potential variants of *W. cuspidata* (Figure 6). Such an outcome is consistent with signal theory predictions where an decrease in Type I errors results in an increase of the probability of making Type II errors (Endler and Mappes, 2017; Lichtenberg et al., 2020) highlighting the benefits of using PDFs to the study of plant mimicry.

Our empirical and statistical evidence partially addresses theoretical positions of pollinator generalization (Fields et al., 1991) and/or generalized food deception in orchids (Jersáková et al., 2006). Considering pollinator decision making, generalization refers to an animal responding to stimuli that differ in some dimension from a target stimulus (Fields et al., 1991; Aguiar et al., 2020). For example, inexperienced honeybees predominantly use simple elemental cues and will generalize to similar shapes (Horridge, 2009) or colors (Giurfa, 2004), whilst experienced bees show evidence of fundamentally different processing like statistical learning enables avoidance of perceptual errors resulting from generalization (Avarguès-Weber et al., 2020). Generalized food deception is a description of how some orchids show evidence of achieving pollination from insect species that may lack a capacity to overcome the limitations of reliable food identification via simple elemental cues (Steiner, 1998; Jersáková et al., 2006). Our understanding of the way these two types of generalization theories may interplay will likely benefit from the formal framework provided by the PDFs (Figure 6), and a better description on how pollinator experience mediates different choice criteria as modeled by the color discrimination functions (Figures 3, 4).

Information from other floral traits, such as shape (Dyer and Chittka, 2004) and scent (Kunze and Gumbert, 2001; Leonard et al., 2011), have been shown to reduce information uncertainty in behavioral experiments and thus could help to set the balance between errors of Type I and II in the presence of color noise in deceptive orchid systems. Indeed, an important caveat in this field of research is that discrimination behavior is modeled based on color differences between matching floral parts. However, other cues are available to pollinators when making choices on visiting flowers. For example, there may be differences in color pattern that could inform foraging decisions, particularly if there is a variable model or multiple model species (e.g., Jersáková et al., 2016; Scaccabarozzi et al., 2018). Outside of color, floral odor (Leonard et al., 2011) and morphology (Howard et al., 2018) are used as cues to identify suitable food sources, and corolla shape has shown to be important for successful mimicry of flowers (Jersáková et al., 2012). More work is needed on how pollinators use colors together with other cues to discriminate between flowers and what this might mean for mimicry systems.

The approaches we present here allow for a more nuanced understanding of floral mimicry systems. Studies evaluating whether a rewardless plant uses a mimetic strategy often involve spectral reflectance measurements that are used to infer whether the mimic can be recognized by pollinators as different from the model based on a simple color discrimination threshold (Peter and Johnson, 2008). However, our use of functions (Equations 1 and 2) coupled with PDFs modeling illustrates that the ability of a pollinator to discern between model and mimic follows a non-linear relationship with color distance (Figures 3, 4), and that the likelihood of successful discrimination is greatly increased by experience foraging on the model/mimic. The use of discrimination functions combined with PDFs for modeling flower color variability represents a new solution for understanding other systems, such as: (i) mimicry of multiple models (Jersáková et al., 2016; Scaccabarozzi et al., 2018), (ii) systems where pollinators show some level of discrimination against the mimic despite effective pollination (e.g., de Jager et al., 2016), (iii) putative cases of generalized mimicry where color resemblance between mimic and model is very low (Jersáková et al., 2006), and (iv) the evolution of flower polymorphism (Kagawa and Takimoto, 2016). For example, our discrimination functions, and PDF of color variability can be used to model discrimination ability by pollinators to variable color based on real data in simulation experiments designed to explain the evolution of color polymorphism at species or population levels (Kagawa and Takimoto, 2016).

Further, this approach can be extended beyond mimicry systems, to understanding the foraging choices of pollinators when faced with a series of rewarding plant species, that may vary in the reward received compared with energy expended. A key challenge, however, for applying this approach will be the lack of knowledge of the psychophysics of pollinators outside of certain model species, although published data currently exists for three key bee pollinators (Garcia et al., 2017), and with these new methods it will be possible to push the frontiers of pollination biology and color signal evolution.

## Data Availability Statement

The datasets generated for this study can be found in online repositories. The names of the repository/repositories and accession number(s) can be found at: https://doi.org/10.6084/m9.figshare.12899816.v1.

## Author Contributions

JG, AD, and RP developed the original conceptual framework of the study. CP collected the spectral data. JG analyzed and modeled the data. All authors contributed to writing and reviewing the manuscript.

## Conflict of Interest

The authors declare that the research was conducted in the absence of any commercial or financial relationships that could be construed as a potential conflict of interest.
